# Artificial Intelligence and COVID-19 Using Chest CT Scan and Chest X-ray Images: Machine Learning and Deep Learning Approaches for Diagnosis and Treatment

**DOI:** 10.3390/jpm11100993

**Published:** 2021-09-30

**Authors:** Roberta Fusco, Roberta Grassi, Vincenza Granata, Sergio Venanzio Setola, Francesca Grassi, Diletta Cozzi, Biagio Pecori, Francesco Izzo, Antonella Petrillo

**Affiliations:** 1 IGEA SpA Medical Division—Oncology, Via Casarea 65, Casalnuovo di Napoli, 80013 Naples, Italy; r.fusco@igeamedical.com; 2 Division of Radiology, Università Degli Studi Della Campania Luigi Vanvitelli, 80138 Naples, Italy; grassi.roberta89@gmail.com (R.G.); francescagrassi1996@gmail.com (F.G.); 3 Italian Society of Medical and Interventional Radiology (SIRM), SIRM Foundation, 20122 Milan, Italy; 4 Division of Radiology, Istituto Nazionale Tumori IRCCS Fondazione Pascale—IRCCS di Napoli, 80131 Naples, Italy; s.setola@istitutotumori.na.it (S.V.S.); a.petrillo@istitutotumori.na.it (A.P.); 5 Division of Radiology, Azienda Ospedaliera Universitaria Careggi, 50134 Florence, Italy; cozzid@aou-careggi.toscana.it; 6 Division of Radiotherapy and Innovative Technologies, Istituto Nazionale Tumori IRCCS Fondazione Pascale—IRCCS di Napoli, 80131 Naples, Italy; b.pecori@istitutotumori.na.it; 7 Division of Hepatobiliary Surgery, Istituto Nazionale Tumori IRCCS Fondazione Pascale—IRCCS di Napoli, 80131 Naples, Italy; f.izzo@istitutotumori.na.it

**Keywords:** COVID-19, computed tomography, X-ray, artificial intelligence, machine learning, deep learning

## Abstract

Objective: To report an overview and update on Artificial Intelligence (AI) and COVID-19 using chest Computed Tomography (CT) scan and chest X-ray images (CXR). Machine Learning and Deep Learning Approaches for Diagnosis and Treatment were identified. Methods: Several electronic datasets were analyzed. The search covered the years from January 2019 to June 2021. The inclusion criteria were studied evaluating the use of AI methods in COVID-19 disease reporting performance results in terms of accuracy or precision or area under Receiver Operating Characteristic (ROC) curve (AUC). Results: Twenty-two studies met the inclusion criteria: 13 papers were based on AI in CXR and 10 based on AI in CT. The summarized mean value of the accuracy and precision of CXR in COVID-19 disease were 93.7% ± 10.0% of standard deviation (range 68.4–99.9%) and 95.7% ± 7.1% of standard deviation (range 83.0–100.0%), respectively. The summarized mean value of the accuracy and specificity of CT in COVID-19 disease were 89.1% ± 7.3% of standard deviation (range 78.0–99.9%) and 94.5 ± 6.4% of standard deviation (range 86.0–100.0%), respectively. No statistically significant difference in summarized accuracy mean value between CXR and CT was observed using the Chi square test (*p* value > 0.05). Conclusions: Summarized accuracy of the selected papers is high but there was an important variability; however, less in CT studies compared to CXR studies. Nonetheless, AI approaches could be used in the identification of disease clusters, monitoring of cases, prediction of the future outbreaks, mortality risk, COVID-19 diagnosis, and disease management.

## 1. Introduction

In December 2019, a large outbreak of a novel coronavirus infection occurred in Wuhan, Hubei Province, China. The novel coronavirus was named severe acute respiratory syndrome coronavirus 2 (SARS-CoV-2) by the International Committee on Taxonomy of Viruses and led to a dramatic pneumonia outbreak in China [1,2,3]. The disease caused by the virus, named coronavirus disease (COVID-19) by the World Health Organization (WHO), can be spread through human-to-human contact. On January 30, 2020, the WHO declared a global public health emergency against the outbreak of COVID-19 [4,5,6].

The COVID-19 diagnosis is confirmed by the positive results of the nucleic acid amplification test of the respiratory tract or blood specimens using reverse transcription real-time fluorescence polymerase chain reaction (RT-PCR) [7,8]. However, methods like chest X-ray (CXR) and chest Computed Tomography (CT) scan are medical imaging techniques, which are widely used to assess the pneumonia due to COVID-19 [9,10,11,12,13,14,15,16,17,18,19]. The reported sensitivity of CXR for COVID-19 pneumonia is relatively low in the early phase of the disease and in mild cases (69%). Conversely, CT shows greater sensitivity for early pneumonic change, disease progression, and alternative diagnosis; the administration of the intravenous contrast medium, is essential for the diagnosis of pulmonary thromboembolism [20,21,22,23,24,25,26,27,28,29,30,31,32,33,34,35,36,37]. Despite recent advances in diagnostic tools, radiologic imaging alone is not sufficient for the COVID-19 pneumonia diagnosis. Imaging should be associated to clinical and laboratory testing. In addition, the American College of Radiology, so as the Italian Society of Radiology (SIRM) does not recommend chest CT as a screening tool, suggesting this method only for symptomatic patients with specific clinical indications. Bilateral distribution of ground glass opacities (GGO) with or without consolidation in posterior and peripheral lungs was the cardinal hallmark of COVID-19 disease [6,22]. Among COVID-19 patients, it is reasonable to assume that those with a very severe disease could exhibit high risk of venous thromboembolism, including deep vein thrombosis and/or pulmonary embolism. In this scenario, it is opened the question on the use of contrast medium during CT studies [20,21,22].

The mathematical models for COVID-19 pandemic, confirmed by practical evidence in China, in Italy, and in the rest of the world, have shown that the rapid substantial increase in the number of critically ill patients exceeds in the total capacity of Intensive care units (ICUs), even excluding routine critical admissions for trauma, stroke, and other emergencies. 

In the context of the COVID-19 outbreak, there is a painstaking need for ready-to-use resources for data acquisition and artificial intelligence (AI) algorithms to accelerate the search for effective and safe treatments. The progressive integration of radiomics approaches and AI-based solutions in healthcare is already changing established paradigms in the entire healthcare ecosystem, leveraging the progressive digitalization of medical data [38,39,40,41,42,43,44,45,46,47,48,49,50,51,52,53]. Specifically, diagnostic and decision support systems developed for medical imaging are the first successful examples of innovation for health. AI-based methods have led to diagnostic applications that accelerate image acquisition, preprocessing, annotation and interpretation, offering an “augmentation” of the radiologists, rather than their unrealistic substitution. In particular, the application of AI in medical imaging has improved the assessment, diagnosis and early detection of neurodegenerative diseases, heart diseases, with a specifically high impact on breast and lung cancer [38,39,40,41,42,43,44,45,46,47,48,49,50,51,52,53].

Deep learning (DL) and machine learning (ML) are branches of AI that focus on producing systems that can learn from examples and improve without being explicitly programmed. ML is the study of computer algorithms that can improve automatically through experience and using data. Machine learning algorithms build a model based on sample data, known as “training data”, to make predictions or decisions without being explicitly programmed to do so. Machine learning algorithms are used in a wide variety of applications, such as in medicine, email filtering, speech recognition, and computer vision, where it is difficult or unfeasible to develop conventional algorithms to perform the needed tasks. A subset of machine learning is closely related to computational statistics, which focus on making predictions using computers; but not all machine learning is statistical learning. The study of mathematical optimization delivers methods, theory and application domains to the field of machine learning. Data mining is a related field of study, focusing on exploratory data analysis through unsupervised learning. 

Deep learning is a class of machine learning algorithms that uses multiple layers to progressively extract higher-level features from the raw input. For example, in image processing, lower layers may identify edges, while higher layers may identify the concepts relevant to a human such as digits or letters or faces. Deep-learning architectures such as deep neural networks, deep belief networks, deep reinforcement learning, recurrent neural networks and convolutional neural networks have been applied to fields including computer vision, speech recognition, natural language processing, machine translation, bioinformatics, drug design, medical image analysis, material inspection and board game programs, where they have produced results comparable to and in some cases surpassing human expert performance.

DL and ML have been applied successfully in many fields, including health care and medical informatics. One important research direction leverages DL and ML to understand and fight COVID-19. Numerous lines of research have been initiated for the application and development of COVID-19-related DL and ML algorithms. 

Several review articles have been published on the use of artificial intelligence approaches in COVID-19 research. Agbehadji et al. [54], summarized how big data and AI models can be used for case detection and contact tracing of COVID-19. Bullock et al. [55], discussed how AI is used to evaluate the challenges of COVID-19 at different scales, including molecular, medical, and epidemiological applications. Naud´e [56] highlighted the actual and potential applications of AI in fighting COVID-19. Wu et al. [57], surveyed the application of big data technology for preventing and managing COVID-19 in China. 

Alballa et al. [58], review the recent ML algorithms in this field and focus on their potential in two main applications: diagnosis of COVID-19 and prediction of mortality risk and severity, using simple clinical and laboratory data; they analyze the main features that were found to be the most relevant to these applications. 

Our aim is to report an overview and update on AI-based methods application in COVID-19 disease using radiological images including CXR and CT focus on their potential in two main applications: diagnosis of COVID-19 and prediction of mortality risk and severity.

This narrative review is the result of an autonomous study without protocol and a registration number. 

## 2. Methods 

### 2.1. Search Criterion 

Several electronic datasets were searched: PubMed (US National Library of Medicine, http://www.ncbi.nlm.nih.gov/pubmed accessed on 24 June 2021), Scopus (Elsevier, http://www.scopus.com/ accessed on 24 June 2021), Web of Science (Thomson Reuters, http://apps.webof knowledge.com/ accessed on 24 June 2021) and Google Scholar (https://scholar.google.it/ accessed on 24 June 2021). The following search criteria have been used: “COVID-19” and “X-ray” and “ARTIFICIAL INTELLIGENCE”; “COVID-19” and “CT” and “ARTIFICIAL INTELLIGENCE”; “COVID-19” and “X-ray” and “DEEP LEARNING”; “COVID-19” and “CT” and “DEEP LEARNING”; “COVID-19” and “X-ray” and “MACHINE LEARNING”; “COVID-19” and “CT” and “MACHINE LEARNING”.

The search covered the years from January 2019 to June 2021. The reference lists of the found papers were analyzed for papers not indexed in the electronic databases. All titles and abstracts were analyzed. The inclusion criteria were studied evaluating the use of AI methods in COVID-19 disease reporting performance results in terms of accuracy or precision or area under Receiver Operating Characteristic (ROC) curve (AUC). Articles published in the English language were included. Exclusion criteria were different topics, unavailability of full text, and not sufficient data.

### 2.2. Statistical Analysis

The summarized accuracy, precision or specificity were calculated in terms of mean, standard deviation value and range. The Chi square test was used to assess differences statistically significant between CXR and CT results. *p* value < 0.05 was considered significant for all tests.

All analyses were performed using IBM SPSS Statistics 24 (IBM, Armonk, NY, USA).

## 3. Results 

**Figure 1:**
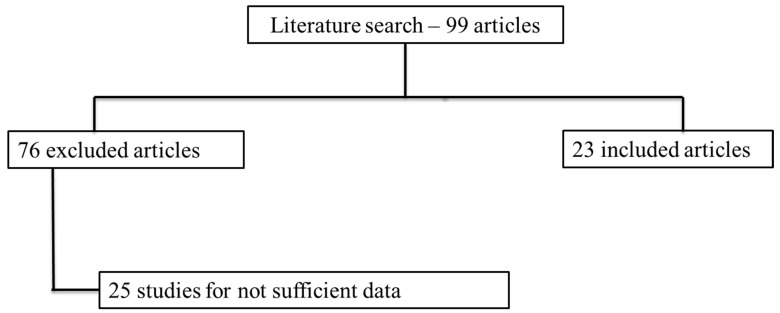
Flowchart of the included papers.

We identified 84 potentially relevant references through electronic searches. We identified 15 references through scanning reference lists of the identified paper that we added to the 84 references previously selected (total number of articles was 99). We then excluded 51 clearly irrelevant articles through screening titles and reading abstracts. We excluded 25 articles for the reasons listed in the exclusion criteria. A total of 23 article met the inclusion criteria. A diagram of included and excluded studies was summarized in the study flow diagram (Figure 1). 

Table 1 reports the classification problem, the classification approach and the performance results of the selected papers.

Thirteen papers using CXR and AI approaches in the COVID-19 disease were identified. The summarized mean value of the accuracy and precision of CRX in COVID-19 disease were 93.7% ± 10.0% of standard deviation (range 68.4–99.9%) and 95.7% ± 7.1% of standard deviation (range 83.0–100.0%), respectively. 

Ten papers using chest CT and AI approaches in the COVID-19 disease were found. The summarized mean value of the accuracy and specificity of CT in COVID-19 disease were 89.1% ± 7.3% of standard deviation (range 78.0–99.9%) and 94.5% ± 6.4% of standard deviation (range 86.0–100.0%), respectively. 

No statistically significant difference in summarized accuracy mean value between CXR and CT was observed using the Chi square test (*p* value > 0.05).

## 4. Discussions

Artificial intelligence approaches have been used to predict the outbreak, to diagnose the disease, to analyze CXR and CT scan images, and more recently to predict mortality or progression risk to severe respiratory failure. This evidence clearly indicates the need for the most rapid and accurate diagnostic and stratification of patients with COVID-19, with technologies and expertise easily accessible from all nodes of the healthcare system with responsibility of diagnosis of COVID-19 and management of patients (either in the health structures or at home) [59]. 

Chest radiographs are first-line investigations in many countries. Researchers could examine not only the initial imaging findings and extent of respiratory involvement, but also how radiographic progression in serial studies correlates with patients’ clinical outcome [60,61,62,66]. CT examination has been used extensively worldwide to evaluate the grade and the extension of the viral pneumonia by COVID-19 and in the follow-up, which are also based on AI algorithms [67,68,69,70]. Several radiological organizations do not recommend CT as primary diagnostic/screening tool for COVID-19 [71,72,73,74] or have excluded CT findings from its diagnostic criteria [75]. Radiologists focus on main CT findings (GGO, consolidation, reticulation/thickened interlobular septa, nodules), and lesion distribution (left, right or bilateral lungs) [76,77,78,79,80]. 

AI methods seek to exploit mainly for characterizing COVID-19 pneumonia CT patterns, for monitoring patients in clinical settings and for estimating efficacy of treatment. Based on the data derived from clinical parameters, AI may provide critical data for resource allocation and decision-making by prioritizing the need of ventilators and respiratory supports in the Intensive Care Unit [81,82,83]. AI was used for the COVID-19 disease detection and quantification from CXR and CT images [63,81,82,83,84,85,86,87,88]. AI can also be used for predicting the chances of recovery or mortality in COVID-19 and to provide daily updates, storage and trend analysis and charting the course of treatment.

CT scan-based and CXR-based identification and detection of COVID-19 have been implemented using pretrained networks such as InceptionV3, VGGNet, InceptionResNetV2, ResNet, etc., and achieved benchmark accuracies as high as 99% [84].

At the same time, radiomics approaches can be usefully implemented, focusing on segmentation techniques of the lung parenchyma based on region growing techniques and on other radiomics COVID-19 specific features and their use with machine learning such as Support Vector Machines (SVMs) or Random Forests [86,87,88].

### 4.1. Application on Chest X-Ray Images

In the study of Sethy et al. [83], the deep learning methodology is reported for detection of a coronavirus infected patient by CXR. The suggested classification model, Resnet50 plus Support Vector Machine (SVM), achieved accuracy and false positive rate of 95.38% and 95.52% respectively for detecting COVID-19. 

Jiao et al. [89], using the CXR as input to an EfficientNet deep neural network combined with clinical data, assessed the ability to predict COVID-19 disease severity (critical or non-critical). They reported that when CXR was added to clinical data for severity prediction, the area under the receiver operating characteristic curve (ROC-AUC) increased from 0.821 to 0.846 on internal testing and from 0.731 to 0.792 on external testing; when deep-learning features were added to clinical data for progression prediction, the concordance index (C-index) increased from 0.769 to 0.805 on internal testing and from 0.707 to 0.752 on external testing; when image and clinical data were combined C-index increase from 0.805 to 0.781 on internal testing and from 0.752 to 0.715 on and internal testing.

Al-Waisy et al. [90], proposed COVID-CheXNet system that is made by combining the results generated from two different deep learning models (e.g., ResNet34 and HRNet) on CXR: two predicted probability scores are computed, and the highest probability score is used to assign the input image to one of two classes for detecting COVID-19. The proposed COVID-CheXNet system reached to diagnose the COVID-19 patients with a detection accuracy rate of 99.99%, a sensitivity of 99.98%, a specificity of 100% and a precision of 100%. Cases used in this study come from different databases: 200 X-ray images with confirmed COVID-19 infections come by Cohen’s GitHub database [91]; 200 COVID-19 CXRs gathered from three different repositories: Radiopaedia dataset [92], Italian Society of Medical and Interventional Radiology (SIRM) [93] and Radiological Society of North America (RSNA) [94]; 400 normal CXR by Kaggle’s CXR dataset [95].

Ozcan et al. [96], proposed single layer-based (SLB) and feature fusion based (FFB) composite systems to detect COVID-19 in X-ray images using deep features. Four types of SLB (including AlexNet-fc6 (SLB1), ResNet18-pool5 (SLB2), ResNet18-fc1000 (SLB3), and ResNet50-fc1000 (SLB4)) and six types of FFB (including fc6-pool5 (FFB1), fc6-fc1000 (FFB2), fc6-fc1000 (FFB3), pool5-fc1000 (FFB4), pool5-fc1000 (FFB5), fc1000-fc1000 (FFB6)) were used in the study. The proposed FFB3 model reached the best average recognition rate of 87.64% in COVID-19, no-finding, and pneumonia classifications while reached as the best average recognition rate of 99.52% in COVID-19 and no-finding classifications.

Ozturc at al. [97] proposed a model for automatic COVID-19 detection using raw CXR images in order to perform the binary classification COVID-19 versus no findings and multi-class classification COVID-19 versus no findings. Their model produced a classification accuracy of 98.08% for binary classes and 87.02% for multi-class cases. The DarkNet model was used in the study as a classifier implementing 17 convolutional layers and introducing different filtering on each layer. 

Du et al. [98], applied machine learning (ML) to the task of detection of SARS-CoV-2 infection using basic laboratory markers. Moreover, they tested ML accuracy adding at laboratory markers the radiologist interpretations of chest radiographs. When they used the combination of laboratory markers and radiologist interpretations, the sensitivity of ML was over 90% while keeping moderate specificity. 

Dey et al. [99], proposed a classifier ensemble technique, utilizing Choquet fuzzy integral. It classifies CXR images in common pneumonia, confirmed COVID-19, and healthy lungs. They utilized the pre-trained convolutional neural network models to extract features and classify the CXR images using two dense layers and one softmax layer. The proposed method provides 99.00%, 99.00%, 99.00%, and 99.02% average recall, precision, F-score, and accuracy, respectively. 

Alruwaili et al. [100], proposed an enhanced Inception-ResNetV2 deep learning model that can diagnose chest X-ray scans with high accuracy of 99.83%. Besides, a Grad-CAM algorithm is used to enhance the visualization of the infected regions of the lungs in CXR images. 

Bukhari et al. [101], employed ResNet50 for COVID-19 detection using CXR images. They tried to differentiate four types of classes, which are healthy normal, bacterial pneumonia, viral pneumonia, and COVID-19 cases. They achieved an average accuracy of 98.18% and a F1-score of 98.19%. 

Khan et al. [102], proposed a model named CoroNet to identify COVID-19 in x-ray and CT scans utilizing a pretrained Xception convolution network. For the four classes (viral pneumonia, COVID-19, bacterial pneumonia, and normal), the first experiment attained an accuracy of 89.6%, while for three classes (normal, COVID-19, and pneumonia) obtained a total accuracy of 95.0%.

A COVIDX-Net model to help radiologists in identifying and diagnosing COVID-19 in CXR images was developed by Hemdan et al. [103]. They compared seven performances of seven pretrained deep learning networks; they are the InceptionV3, MobileNetV2, VGG19, DenseNet201, Inception-ResNetV2, ResNetV2, and Xception model. Based on their experiments, the VGG19 model achieved the highest accuracy of 90%. 

Sethy and Behera [83], introduced a hybrid approach that utilizes deep learning for feature extraction and support vector machine (SVM) for detecting patients contaminated with COVID-19 by using CXR images. Using the pretrained 13 distinct Convolutional Neural Network models, the SVM provided the best results on the deep features of the ResNet50 model achieving accuracy of 95.38% for detecting COVID-19 (ignoring SARS, MERS and ARDS).

Ouchicha et al. [104], proposed a model named CVDNet to diagnose the COVID-19 cases. This model employed local and global features of CXR by using two parallel layers with various kernel sizes reaching an average accuracy of 97.20% for detecting COVID-19 cases. 

### 4.2. Application on Chest CT images

Gozes et al. [81], used deep learning models to explore AI CT image analysis tools in the detection, quantification, and tracking of coronavirus. A total of 106 COVID-19 chest CT scans (50 labeled by a radiologist, and other 56 by RT-PCR test) and 99 normal ones were used to find potential COVID-19 thoracic CT features and to evaluate disease progression over time, generating a quantitative score. Utilizing the deep-learning image analysis system developed, they achieved classification results for COVID-19 versus no COVID-19 by chest CT of 0.948 of AUC (95%CI: 0.912–0.985).

Proof of principle of diagnostic capability of deep learning methods using CT images to detect COVID-19 disease have been demonstrated by Wang et al. [63] on 1119 CT images of pathogen-confirmed COVID-19 cases versus typical viral pneumonia. Their internal validation achieved a total accuracy of 89.5% with a specificity of 0.88 and sensitivity of 0.87. The external testing dataset showed a total accuracy of 79.3% with a specificity of 0.83 and sensitivity of 0.67. 

Li et al. [85], investigated a deep learning model, COVID-19 detection neural network (COVNet), by extraction of visual features from volumetric chest CT images to detect COVID-19. The datasets were collected from six hospitals between August 2016 and February 2020. The sensitivity and specificity for detecting COVID-19 was 114 of 127 (90% [95% CI: 83%, 94%]) and 294 of 307 (96% [95% CI: 93%, 98%]), respectively, with an AUC of 0.96 (*p*-value < 0.001). 

Ko et al. [105], investigated a simple 2D deep learning framework, and named the fast-track COVID-19 classification network (FCONet), in order to diagnose COVID-19 pneumonia based on a single chest CT image. FCONet was developed by transfer learning using one of four state-of-the-art pretrained deep learning models (VGG16, ResNet-50, Inception-v3, or Xception) as a backbone. Among the four pretrained models of FCONet, ResNet-50 showed excellent diagnostic performance (sensitivity 99.58%, specificity 100.00%, and accuracy 99.87%) and outperformed the other three pretrained models in the testing data set. In the additional external testing data set using low-quality CT images, the detection accuracy of the ResNet-50 model was the highest (96.97%). 

Nguyen et al. [106], examined deep learning models in order to identify COVID-19-positive patients on 3D CT datasets from different countries. The models achieved accuracy/AUC values of 0.87/0.826 (dataset at UT Southwestern), 0.97/0.988 (dataset at China), and 0.86/0.873 (dataset at Iran). 

Zhang et al. [64], used artificial intelligence technology proposing a COVSeg-NET model that can segment GGO lesions in COVID-19 chest CT images. The COVSeg-NET model is based on the fully convolutional neural network model structure, which mainly includes convolutional layer, nonlinear unit activation function, maximum pooling layer, batch normalization layer, merge layer, flattening layer, sigmoid layer, and so forth. The results showed a sensitivity and specificity of the COVSeg-NET model of 0.447 and 0.996 respectively. 

Song et al. [107], developed a deep learning network, which is called DeepPneumonia, to diagnose COVID-19 cases analyzing CT scans. Their proposed system was built on the ResNet50 using transfer learning technology. It could localize the essential lesion characteristics, especially GGO. Their system achieved an average AUC of 0.99 and sensitivity score of 93%. Besides, it reached an average AUC of 0.95 and sensitivity of 96% for bacterial pneumonia-infected cases. 

Wang et al. [65], developed an artificial intelligence system in a time-to-event analysis framework to integrate chest CT and clinical data for risk prediction of future deterioration to critical illness in patients with COVID-19. The artificial intelligence system achieved a C-index of 0.80 for predicting individual COVID-19 patients as having critical illness, and successfully stratified the patients into high-risk and low-risk groups with distinct progression risks (*p* < 0.0001).

Xu et al. [108], proposed a fully automated COVID-19 diagnosis based on a 3D deep learning network-using chest CT scans. Their proposed system consists of four basic stages, which are pre-processing, candidate region segmentation, classification for each candidate region, and overall infection probability. The experimental results of this study showed that the summarized accuracy rate was 86.7%. 

### 4.3. Critical Considerations and Conclusions

In addition, if the summarized accuracy of the selected papers is high, there was an important variability. The accuracy and applicability of AI approaches in COVID-19 from CXR or chest CTs have questioned, based on concerns of the radiologists’ association, and given the impact of selection bias reported in first published results. Moreover, the limitation of this methodology is that if the patient is in a critical situation and unable to attend for CXR or CT scanning.

The analyzed papers showed the great potential of AI in COVID-19 pandemic by helping complex decision-making. However, most of the analyzed papers were experimental, and the produced models have not been deployed in real-world clinical setting. Those reported are impeded by several limitations. The available data sets may suffer from selection bias. The prognosis studies mostly encompass inpatients, who are usually sicker, whereas the diagnosis studies typically involve patients who already exhibit symptoms fitting with COVID-19. More data are needed on asymptomatic individuals and those with mild symptoms, who might not visit the hospital. Moreover, most of the studies reviewed employed imbalanced data sets, that is, those where many records in the training data set represent the negative class, and the positive class is under-represented. Thus, the reported performance of various AI algorithms applied in this context may have been affected by polarization of the context: a pandemic scenario. A high accuracy value in such cases could be attributed to the ability of the model to accurately identify negative samples and erroneously exclude all the positive COVID-19 cases. More effort is required to handle imbalanced data sets prior to the application of AI to COVID-19. The predictive performance of the models might also differ when using representative data that incorporates the targeted population, which merits further investigation.

Moreover, although AI is a promising tool in precision medicine, many factors such as low signal-to-noise ratio and complex data integration have challenged its efficacy. Both CXR and CT showed a high accuracy to detect pneumonia by COVID-19 and to predict the disease evolution, but which CXR is the first examination in this context and thus more data is available, CT is more capable to investigate extension and critical issues of the disease. However, CT images represent a difficult classification task due to the relatively large number of variable objects, specifically the imaged areas outside the lungs that are irrelevant to the diagnosis of pneumonia. Notably, the assessed features of the CT images were from patients with severe lung lesions at later stages of disease development. A larger number of databases to associate this with the disease progress and all pathologic stages of COVID-19 are necessary to optimize the diagnostic system.

In conclusion, AI approaches could be used in the identification of disease clusters, monitoring of cases, prediction of the future outbreaks, mortality risk, diagnosis of COVID-19, disease management by resource allocation, facilitating training, record maintenance and pattern recognition for studying the disease trend. 

## Figures and Tables

**Table 1:** Imaging Modality, Dataset, classification problem, classification approach and performance results of the selected papers.

Authors	Methodology	Imaging Modality	Dataset	Limits	Classification Problem	Classification Model	Accuracy [%]	Specificity [%]	Precision [%]	Area Under ROC Curve
Sethy et al.	deep learning	CXR	Firt dataset: 25 number of COVID-19+ and 25 number of COVID-19- X-ray images. Second dataset: 133 X-ray images of COVID-19+, including MERS, SARS, and ARDS and 133 chest X-ray images as COVID-19-	Number of patients. Moreover, the limitation of this methodology is that if the patient is in a critical situation and unable to attend for CXR scanning. The model is limited to classify the input chest X-ray image into only two classes either normal or COVID-19	COVID-19 detection	resnet50 plus Support Vector Machine	95.38			
Jiao et al.	deep learning	CXR	1834 patients were identified and assigned to the model training (*n* = 1285), validation (*n* = 183), or testing (*n* = 366) sets. The number of patients that were identified for external testing of the model were 475.	The artificial intelligence model showed decreased performance on the external testing set relative to the internal testing set, indicating that generalization might not be possible. This finding could have been due to several factors, including heterogeneous data and image acquisition between the different hospital systems. The model is limited to classifying the input chest X-ray image into only two classes either normal or COVID-19	predict the binary outcome of COVID-19 disease severity (critical or non-critical)	EfficientNet deep neural network and clinical data				0.85 on internal testing and 0.80 on external testing
Al-Waisy et al.	deep learning	CXR	200 X-ray images with confirmed COVID-19 infection come by Cohen’s GitHub database [58]; 200 COVID-19 CXR gathered from three different repository: Radiopaedia dataset [59], Italian Society of Medical and Interventional Radiology (SIRM) [60], and Radiological Society of North America (RSNA) [61]; 400 normal CXR by Kaggle’s CXR dataset [62]	Cases used in this study come from different databases. The model is limited to classifying the input chest X-ray image into only two classes, either normal or COVID-19	COVID-19 detection	COVID-CheXNet system made by combining the results generated from two different deep learning models	99.99	100	100	
Ozcan et al.	deep learning	CXR	127 X-ray images diagnosed with COVID-19, 500 no-findings and 500 pneumonia class	No external testing of the model and the patient number is low for multi class classification	COVID-19 versus no findings classification/multi-class classification COVID-19 versus no findings versus pneumonia	single layer-based (SLB) and a feature fusion based (FFB) composite systems using deep features	99.52/87.64		98.03/99.7	
Ozturk et al.	deep learning	CXR	127 X-ray images diagnosed with COVID-19, 500 no-findings and 500 pneumonia class	No external testing of the model and the patient number is low for multi class classification	COVID-19 versus no findings classification/multi-class classification COVID-19 versus no findings versus pneumonia	DarkNet model implementing 17 convolutional layers and introducing different filtering on each layer	98.08/87.02		98.03/89.96	
Du et al.	machine learning	CXR	447 cases with COVID-19; 405 with other viral PNA, 1515 with bacterial PNA, 1862 with Clinical PNA, 256 with other infections and 663 with other diseases	The model has a moderate specificity	COVID-19 detection		68.4			
Dey et al.	classifier ensemble technique	CXR	A total of 506 viral lung infection cases including 468 cases with COVID-19;46 bacterial lung infection and 26 fungal lung infection by https://github.com/ieee8023/covid-chestxray-dataset.; 1583 normal CXR and 4273 COVID-19+ CXR by https://www.kaggle.com/paultimothymooney/chest-xray-pneumonia/version/2.	No external testing of the model and patient number.	classification of Normal, COVID-19, and Pneumonia cases	Choquet fuzzy integral using two dense layers and one softmax layer	99.02		99	
Alruwaili et al.	deep learning	CXR	2905 CXR images, which are distributed into 219 COVID-19 images, 1345 viral pneumonia images, and 1341 for normal category	No external testing of the model and patient number.	COVID-19 vs. normal vs. viral pneumonia classification	Inception-ResNetV2 deep learning model	99.83		98.11	
Bukhari et al.	deep learning	CXR	93 CXR which have no radiological abnormality; 96 CXR with the radiological features of pneumonia different from COVID-19 infection; 89 digital images of chest X-rays of patients diagnosed with COVID-19 infection	The model takes a roughly higher training and testing run time compared to other models due to the complex structure of the inside modules.	healthy normal, bacterial pneumonia, viral pneumonia, and COVID-19 classification	resnet50	98.18		98.14	
Khan et al.	deep learning	CXR	1203 normal, 660 bacterial Pneumonia and 931 viral Pneumonia cases	Small prepared dataset which indicates that given more data, the proposed model can achieve better results with minimum pre-processing of data	viral pneumonia, COVID-19, bacterial pneumonia, and normal classification/normal, COVID-19, and pneumonia classification	CoroNet: pretrained Xception convolution network	89.6/95.0			
Hemdan et al.	deep learning	CXR	25 normal cases and 25 positive COVID-19 images	The model is limited to classifying the input chest X-ray image into only two classes, either normal or COVID-19. Another limit is the number of patients	COVID-19 detection	InceptionV3, MobileNetV2, VGG19, DenseNet201, Inception-ResNetV2, ResNetV2, and Xception model	90		83	
Sethy and Behera	deep learning and machine learning	CXR	25 normal cases and 25 positive COVID-19 images	The model is limited to classifying the input chest X-ray image into only two classes, either normal or COVID-19	detecting COVID-19 (ignoring SARS, MERS and ARDS)	deep learning for feature extraction and support vector machine (SVM) for classification	95.38			
Ouchicha et al.	deep learning	CXR	219 COVID-19, 1341 normal and 1345 viral pneumonia	The model has been trained on a small dataset of few images of various COVID-19, viral pneumonia and normal cases from publically available database	classification of Normal, COVID-19, and Pneumonia cases	local and global features of CXR using two parallel layers with reaching various kernel sizes	97.2			
Gozes et al.	deep learning	CT	106 COVID-19 chest CT scans and 99 normal ones	Patient number is low	COVID-19 versus no COVID-19	robust 2D and 3D deep learning models				0.948
Wang et al. [63]	deep learning	CT	740 for COVID-19 negative and 325 for COVID-19 positive	Sample size was relatively small	COVID-19 versus no COVID-19	GoogleNet Inception v3 convolution neural network	89.5	88		
Li et al.	deep learning	CT	1292 with COVID-19, 1735 for community-acquired pneumonia, and 1325 for non-pneumonia abnormalities	The model is limited to classifying the input chest X-ray image into only two classes, either normal or COVID-19	COVID-19 versus no COVID-19	resnet50		90		0.96
Ko et al.	deep learning	CT	1194 chest CT COVID-19 images and 1357 chest CT images with non–COVID-19 pneumonia	The model is limited to classifying the input chest X-ray image into only two classes, either normal or COVID-19	Classification of COVID-19 patients	FCONet developed by transfer learning using one of four state-of-the-art pretrained deep learning models (VGG16, ResNet-50, Inception-v3, or Xception)	99.87	100		
Nguyen et al.	deep learning	CT	101 with COVID-19, 118 with common Pneumonia and 118 for non-pneumonia abnormalities	Patient number is low for three classes classification	normal, COVID-19, and pneumonia classification	convolutional neural network	87			0.83
			1544 with COVID-19, 1556 with common Pneumonia and 118 for non-pneumonia abnormalities				97			0.99
			281 with COVID-19 and 1068 for non-pneumonia abnormalities				86			0.87
Zhang et al. [64]	CT	CT	There were 406 clearer COVID-19-positive lung CT images. The marked areas in the mask images are 0-“ground glass opacity,” 1-“consolidations,” 2-“lungs other,” 3-“background.	The complexity of the model and the number of patients	segment ground glass opaque lesions in COVID-19 lung CT images	COVSeg-NET model is based on the fully convolutional neural network model structure, which mainly includes convolutional layer, nonlinear unit activation function, maximum pooling layer, batch normalization layer, merge layer, flattening layer, sigmoid layer, and so forth		100		
Song et al.	deep learning	CT	A total of 88 patients diagnosed with the COVID-19, 101 patients infected with bacteria pneumonia, and 86 healthy persons	Patient number is low for a three classes classification	normal verus COVID-19 classification/discriminating COVID-19 patients from others	resnet50		96/86		0.99/0.95
Wang et al. [65]	machine learning	CT	A total of 1051 patients with RT-PCR confirmed COVID-19 and chest CT was included in this study	Patient selection bias, retrospective and multi-institutional nature of the study.	for prediction of COVID-19 progression using CT imaging and clinical data		78		80	
Xu et al.	deep learning	CT	A total of 618 CT samples were collected: 219 samples from 110 patients with COVID-19 and 224 samples from 224 patients with influenza-A viral pneumonia (IAVP)	Patient selection bias, patient number is low	early screening model to distinguish COVID-19 from IAVP and healthy cases through pulmonary CT images	3D deep learning network that consists of four basic stages, which are pre-processing, candidate region segmentation, classification	86.7			

## Data Availability

All data are reported in the manuscript.
